# High levels of drug resistance in commensal *E. coli* in a cohort of children from rural central India

**DOI:** 10.1038/s41598-019-43227-1

**Published:** 2019-04-30

**Authors:** Manju Raj Purohit, Lars Falkdalen Lindahl, Vishal Diwan, Gaetano Marrone, Cecilia Stålsby Lundborg

**Affiliations:** 1 Department of Public Health Sciences, Global Health- Health Systems and Policy (HSP): Medicines focusing antibiotics, Karolinska Institutet, Stockholm, Sweden; 2 Department of Pathology, R.D. Gardi Medical College, Ujjain, India; 3 International Centre for Health Research, Ujjain Charitable Trust Hospital and Research Centre, Ujjain, India; 4 Department of Public Health and Environment, R.D. Gardi Medical College, Ujjain, India

**Keywords:** Epidemiology, Antibiotics

## Abstract

The world is experiencing crisis of antibiotic resistance not only in pathogenic but also in commensal bacteria. We determine the prevalence of antibiotic resistance in commensal *Escherichia coli* in young children in rural setting of central India and search for its correlations with demographic and behavioral factors. At seven time points during a period of 2 years we collected stool samples from 125 children; aged 1–3 in a rural area of Madhya Pradesh. We isolated six isolates of *E. coli* per stool sample and subjected them to antibiotic susceptibility testing. We found resistance to ampicillin, quinolones, cephalosporins, sulfamethoxazole, co-trimoxazole, in at least one isolate from 89% to 100% of children. Extended spectrum beta lactamase producing *E. coli* were identified in all but one child and multidrug resistance was identified in isolates from all children. Female gender (*p* = 0.04) and higher wealth (*p* = 0.03) was significantly correlated with less antibiotic resistance. Thus, the high prevalence of antibiotic resistance in commensal *E. coli* in rural community from India needs urgent measures to control the growing antibiotic resistance crisis.

## Introduction

With the alarming emergence of multidrug resistant strains of various bacteria, antibiotic resistance has become a global public health threat. Resistant strains are not only prevalent in hospital settings but also spread to communities. *Escherichia coli*, a near-ubiquitous colonizer of the gastrointestinal tract may play a crucial role in the spread of resistance within a community^1^ because faecal flora serves as the reservoir of antibiotic resistance genes. Exposure of commensals such as *E. coli* to antibiotics increases the carriage levels of resistant organisms and resistance might be transmitted to a more virulent acquired organism^2^. Animals, humans and the environment including water sources serve as natural habitats of virulent strains of *E. coli*. *E. coli* have been utilized as a precise indicator for the surveillance and spread of antimicrobial resistance among pathogens^3^. The World health Organization (WHO) named *E. coli* as one of nine pathogens deemed to be of the highest concern globally in terms of antibiotic resistance^4^. We have previously seen that commensal *E*. *coli* from apparently healthy children, animal, and water were resistant to many antibiotics^5^.

The emergence and spread of antibiotic resistance is multi-factorial, including existing practices of healthcare providers, patterns of antibiotic use, perception of various stakeholders in the community, over-the-counter availability, socio demographic factors, and safe water and sanitation practices. All these factors influence the health-seeking behaviour of an individual. In India, health-seeking behaviour often begins as home care, traditional healers practice and the informal system with inappropriate use of medicine including antibiotics, an important contributing factor to the antibiotic resistance crisis^6–9^. It has been found that the biophysical and socio behavioural environment, community exposures and ambulatory care have a large impact on acquisition of antibiotic resistance and its transmission^10,11^. However, the majority of existing information on antibiotic resistance pattern has been obtained from hospitalized patients, rather than from samples of community-dwelling persons.

Despite the obvious high burden of infectious diseases^12,13^ and the high prevalence of antibiotic resistance in India, very few studies have described the significance of socio-economic and demographic factors for its contribution for acquisition of antibiotic resistance. These analyses are worthwhile in ascertaining risk factor profiles for designing better target interventions. A follow-up of children (who are less frequently affected by confounding factors such as occupation, travel, and medical history) may help to identify such societal factors for developing context-specific strategies for an effective antibiotic stewardship programme. Thus, we aimed to determine the prevalence of antibiotic resistance of commensal *E. coli* bacteria over a 2-year period in a cohort of children aged 1–3 years in a rural setting in central India and to investigate possible relation between the prevalence of antibiotic resistance and the recorded demographic and behavioural factors of the children’s families.

## Materials and Methods

### Study setting

The study was conducted in Ujjain District, Madhya Pradesh, India. Madhya Pradesh is the second largest state in India^14^ with a population of 72.6 million, with approximately 75% of the population live in rural areas^14^. Six of 60 villages were selected for the study from the Demographic Surveillance Site of Ruxmaniben Deepchand Gardi Medical College (RDGMC). The villages were selected with the following criteria; (a) <5 km from the central village, (b) >500 inhabitants, (c) >15 children of the desired age group (1–3 years old), and (d) <45 minutes transport time to the central research laboratory (CRL) at RDGMC, where all samples were analyzed.

### Sampling population

The detail of sampling population is previously presented^15^. In brief, families were initially surveyed for demographic characteristics (age, sex). A cohort of 125 children aged 1–3 was then randomly selected (using random number tables) from families having lived in the villages for the past one year, planning to do so for the coming three years, and who were willing to participate in the study. No further exclusion criteria were used and no family declined participation. One child was selected from each family. If there were more than one child of the correct age in a family, the youngest child was selected for the study. In the families of the selected cohort, a comprehensive interview was conducted to gather detailed information, including education, profession, and household- and agricultural practices.

### Sample collection and methodology

Samples for microbial analysis were taken from the participating children’s stool at seven time-points during a 2-year period (August 2014–September 2016) at an interval of four months. The stool was collected using a sterile polyethylene sheet. Collected stool was then transferred to a sterile plastic container using a sterile spoon. The stool collection equipment was distributed to the households and caregivers were given instructions on the procedure to collect stool on the morning of the day of sample collection. These instructions were repeated on every subsequent visit. Samples were transported to the CRL at RDGMC on the day of collection. Transport time was <45 minutes and a cold-chain of 4–6 °C was maintained with all samples.

*E. coli* were isolated after plating samples on selective and differential HiCrome^®^coliform chromogenic agar (HiMedia laboratories Pvt. Ltd., Mumbai, India). Six *E. coli* colonies were picked, processed, confirmed with PCR, purified and stored for further analysis as detailed previously^15^. All six confirmed *E. coli* isolates of every sample were subjected to antibiotic susceptibility testing (AST) by the Kirby-Bauer disc diffusion method^16^, using disc strengths based on the recommendations by the Clinical and Laboratory Standards Institute (CLSI)^17^. The isolates were tested against 18 different antibiotics or combinations of antibiotics presented in Table 1 (all acquired from HiMedia laboratories Pvt. Ltd., Mumbai, India). The antibiotics were chosen based on local practice of antibiotic use for treatment of infections by gram-negative coliforms and the current guidelines from CLSI^17^. Colistin resistance was tested by both detecting mcr-1 gene and by disc diffusion only for the three last time points. Result from the AST was recorded in the form of mono-resistance (resistance to a single antibiotic) and multidrug resistance (MDR) (defined as resistance to at least one antibiotic in three or more antibiotic classes). Occurrence of extended spectrum β-lactamase (ESBL) was determined according to CLSI guidelines by the combined disc diffusion method using Muller Hinton agar with cefotaxime (30 μg), cefotaxime/clavulanic acid (30/10 μg), ceftazidime (30 μg), and ceftazidime/clavulanic acid (30/10 μg)^17^. *E. coli* reference strain ATCC 25922 was used for quality control.

**Table 1:** Antibiotics used in the study and their respective class.

Name	Class
Amikacin	*Aminoglycosides*
Ampicillin	*Penicillins*
Cefepime	*4th generation cephalosporin*
Cefotaxime	*3rd generation cephalosporin*
Cefotaxime/Clavulanic acid	*3rd generation cephalosporin/β-lactamase inhibitors*
Ceftazidime	*3rd generation cephalosporin*
Ceftazidime/Clavulanic acid	*3rd generation cephalosporin/β-lactamase inhibitors*
Ciprofloxacin	*2nd generation fluoroquinolone*
Colistin	*Polymyxins*
Co-Trimoxazole (Trimethoprim/sulfamethoxazole)	*Folatepathway inhibitors*
Gentamycin	*Aminoglycosides*
Imipenem	*Carbapenems*
Meropenem	*Carbapenems*
Nalidixicacid	*Synthetic quinolone*
Nitrofurantoin	*Nitrofuran derivatives*
Sulfamethoxazole (Sulphamethiazole)	*Sulfonamides*
Tetracycline	*Tetracyclines*
Tigecycline	*Glycylcyclines*

Considering the same socio-behavioural characteristics of the selected six villages, *E. coli* isolates from one village with least literacy and living standard as described in detail previously^5^ were selected from seven-point time for pathotype and phylogenetic grouping. *E. coli* pathotypes EHEC (*stx1* or/and *stx2* and *eaeA);* EPEC (*eaeA* without *stx1* or *stx2);* STEC (*stx1*or/and *stx2*)*;* EIEC (*ipaH*)*;* EAEC (*aggR*)*; ETEC* (*StIb* or/and *LtI*)*;* DEC (*daaE*)*;* ExPEC (two or more of *papA* and/or *papC*; *afa/dra*; *kpsMT II*; *iutA*; and *sfa/foc*) genes were amplified and identified with previously-described primers by PCR after alkaline lysis DNA extraction^18,19^. The phylogenetic grouping of similar *E. coli* isolate was performed based on *chuA*, *vjaA*, and *TspE4C2* genes which were amplified by PCR with the primer sequences as described in detail elsewhere^20^. All of the amplified PCR products were visualized using a gel documentation system for all *E. coli* isolates.

### Statistical analyses

Data was analyzed using SPSS (SPSS Inc, Chicago, IL, USA) and Excel (Microsoft Corp., Redmond, WA, USA). Descriptive statistics include mean, standard deviation and median for the categorical variables and hypothesis testing, regression models and longitudinal analysis were used for inferential statistics, as appropriate. AST was analyzed both as categorical interpretation of resistance (resistant, intermediate, and susceptible), and directly as numerical variables using the measured inhibitory zone. Correlation of results from the AST with data from the demographic and behavioral survey was done using two-tailed t-tests assuming unequal variance (Welch’s t-test) and linear regression. Resistance to antibiotics was used as dependent variables, and different demographic variables such as age, sex, and different household practices as independent variables. *P-*values less than 0.05 were considered significant.

### Ethical considerations

The collection of data was completed only after having written informed consent from a parent and/or legal guardian for study participation and did not involve any invasive or otherwise harmful procedures. Information about the study and its aims was provided to caregivers in their language. A referral to health facilities in collaboration with the RDGMC was offered if a child needed medical attention. Ethical review number: 2013/07/17-311 (Institutional Ethics committee RDGMC, Ujjain, India). All methods were performed in accordance with the relevant guidelines and regulations.

## Results

In total, 125 children (57 female and 68 male participants) were included in this study. Half (n = 62) of the children were aged 1 year, and 63 children were aged 2–3 years. A summary of selected socio demographic characteristics of the families of the children is presented in Table 2.

**Table 2:** Socio-demographic characteristics of the families of the children (n = 125) included in the study.

Variable	Frequency (%)	Mean	Median
**Number of family members**	906 (100)	7.25	7
Adults (aged 16+)	421 (46.5)	4.30	4
Children aged 0–1	116 (12.8)	0.93	1
Children aged 1–3	177 (19.5)	1.42	1
Children aged 4–15	192 (21)	1.54	1
**Number of animals**	458 (100)	3.66	3
Cow/buffalo	332 (72.5)	2.66	2
Goat	78 (17)	0.62	0
Chicken	41 (9)	0.33	0
Dog	7 (1.5)	0.056	0
**Variable**	**Frequency (%)**	**Variable**	**Frequency (%)**
Family type*		**Main source of drinking water**	
Nuclear	38 (30.4)	Handpump	53 (42.4)
Joint	87 (69.6)	Unprotected dug well	30 (24)
**Education of head of family**		Public tap/standpipe	24 (19)
Uneducated	55 (44)	Tube well/borehole in house	8 (6.4)
Primary (1^st^ to 5^th^ grade)	44 (35)	Piped water to agricultural land	5 (4)
Middle (5^th^ to 8^th^ grade)	16 (12.8)	Piped water into home	3 (2.4)
Secondary (9^th^ to 12^th^ grade)	9 (7)	Protected dug well	1 (0.8)
Tertiary (university)	1 (0.8)	Tanker truck	1 (0.8)
**Occupation of head of family**		**Water purification method**	
Farmer	78 (62.4)	Plastic/steel filter	101 (80.8)
Labor	23 (18.4)	Strain through cloths	20 (16)
Service	3 (2.4)	No purification	4 (3)
Government job	1 (0.8)	**Toilet facility**	
Household work	10 (8)	No facility/bush/field	97 (77.6)
Unemployed	10 (8)	Pit latrin with slab	23 (18.4)
Type of house**		Flush toilet	4 (3)
Kutcha	60 (48)	Ventilated improved pit latrine	1 (0.8)
Pucca	14 (11)	**Substance for cleaning hands**	
Semi-pucca	51 (40.8)	Soap	99 (79)
**Animals in household**		Mud	17 (13.6)
Yes	99 (79)	Ash	5 (2.4)
No	26 (20.8)	Only water	4 (3)

In brief, more than two-thirds of the families were joint rather than nuclear families (nuclear families consist of children and their parents only, whereas joint families are in general larger and can include grandparents, spouses of children, grandchildren, and other relatives; several generations often live together in joint families). The families had a median of seven family members, comprising four adults and three children. Four of five families had livestock, with the majority of livestock being cows/buffalos. The education level was low, with 44% of heads of the families having no education and a further 35% only having primary education. Farming was the most common occupation of the head of the family, followed by labour. About half of the homes of the families were of the so-called–‘Kutcha’ type; these house are mainly built using natural materials such as bamboo, mud, and reeds and have a mud floor. The so-called ‘Pucca’ houses on the other hand are built using materials such as stones, bricks, and cement. ‘Semi-Pucca’ houses have walls and a roof similar to ‘Pucca’ houses but have a mud floor. Almost one in four families had an unprotected dug well as their main source of drinking water.

### Antibiotic susceptibility testing

**Figure 1:**
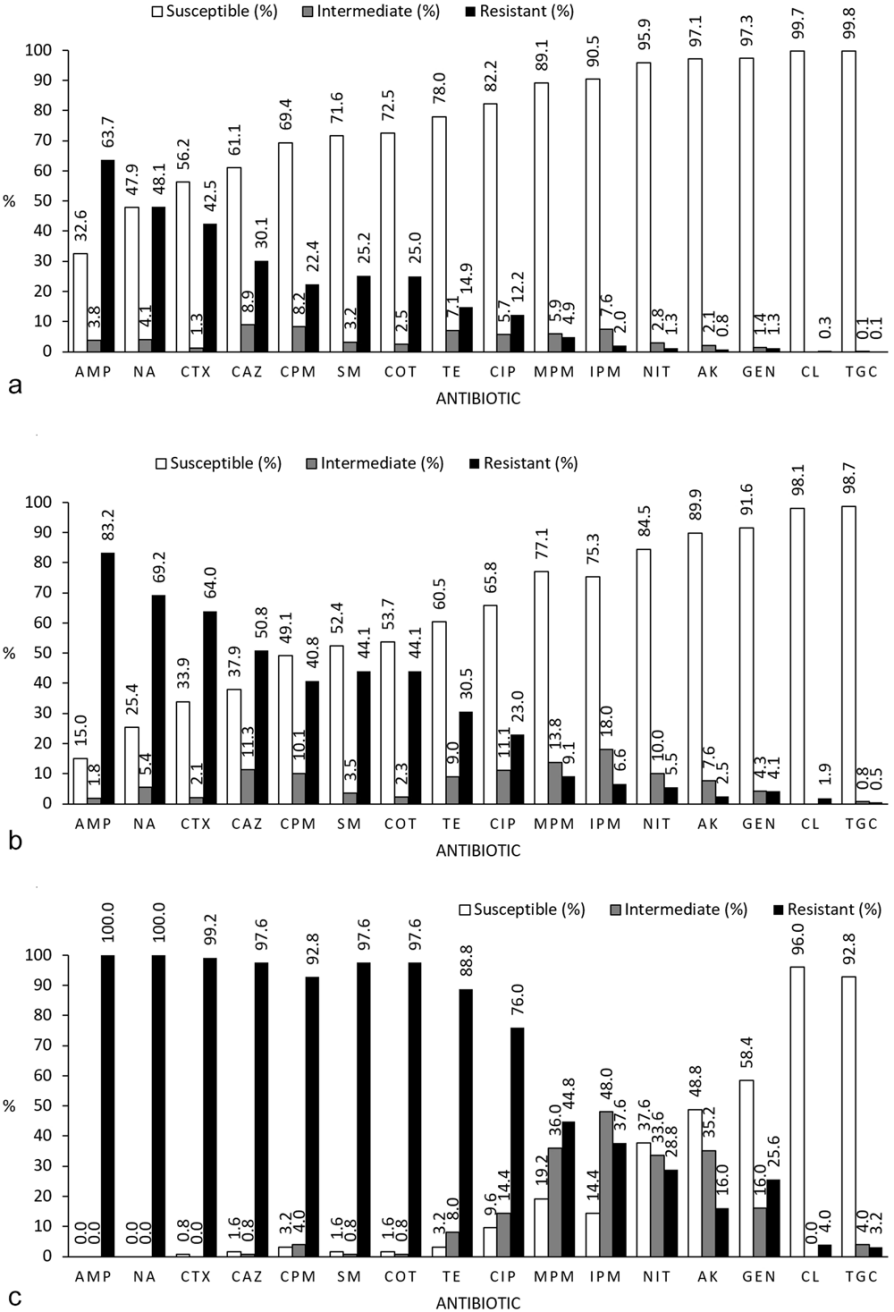
Percentage (%) of antibiotic resistance per (**a**) isolates (n = 4764), (**b**) stool samples (n = 799). The result was considered susceptible if all 6 isolates from the stool sample was found to be susceptible, intermediate if one or more isolates were intermediate resistant (but not resistant), and resistant if one or more isolates was resistant. (**c**) Percentage (%) of antibiotic resistance per child (n = 125) for each antibiotic tested. The result was considered susceptible if all isolates (6 isolates per stool sample and 7 stool samples = 42 isolates per child) from the child was found to be susceptible, intermediate if one or more isolates were intermediate resistant (but not resistant), and resistant if one or more isolates was resistant. AMP: Ampicillin, NA: Nalidixic acid, CTX: Cefotaxime, CAZ: Ceftazidime, CPM: Cefepime, SM: Sulfamethoxazole, COT: Co-trimoxazole, TE: Tetracycline, CIP: Ciprofloxacin; MPM: Meropenem, IPM: Imipenem, NIT: Nitrofurantoin, AK: Amikacin, GEN: Gentamycin, CL: Colistin, TGC: Tigecycline.

A total of 4,764 isolates from 799 stool samples of the 125 children included in this study were analysed using AST. The obtained prevalence of antibiotic resistance per isolate is presented in Fig. 1a. The highest rate of resistance was found for ampicillin; 63.7% of all isolates tested showed ampicillin resistance. In addition, 3.8% of isolates showed intermediate resistance. For meropenem, the rate of resistance was 4.9%, and the rate of intermediate resistance was 5.9%; for imipenem, the rate of resistance was 2.0%, and the rate of intermediate resistance was 7.6%. As expected, the lowest rates of resistance were found for colistin (0.3%) (as determined using mcr-1 gene detection) and tigecycline (0.1% plus 0.1% intermediate). ESBL was present in 37.8% of isolates, and MDR was detected in 49.0% of isolates.

Figure 1b presents the rates of resistance per stool sample, which were calculated using data from all six isolates per stool sample. In 83.2% of stool samples, at least one isolate was found to be resistant to ampicillin, with 1.8% showing intermediate resistance. Meropenem and imipenem resistance was detected in 9.1% (and intermediate resistance in 13.8%) and 6.6% (plus intermediate resistance in 18.0%) of stool samples, respectively. Colistin resistance was found in 0.6% of stool samples, and tigecycline resistance was found in 0.5% of stool samples (plus intermediate resistance in 0.8%). ESBL was present in 59.8% of stool samples, and MDR was detected in 74.1% of stool samples (data not shown).

**Figure 2:**
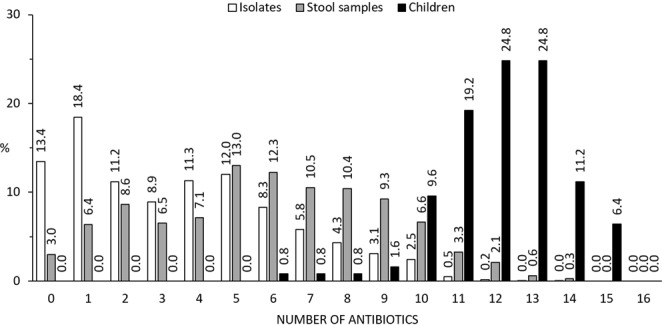
Percentage of non-susceptible (resistant or intermediate) results for isolates (n = 4764), stool samples (n = 799), and children (n = 125). With stool samples, the result is counted as non-susceptible to an antibiotic unless all isolates from the stool sample was determined to be susceptible. With children, the result is counted as non-susceptible to an antibiotic unless all isolates of all stool samples from the child were determined to be susceptible.

**Figure 3:**
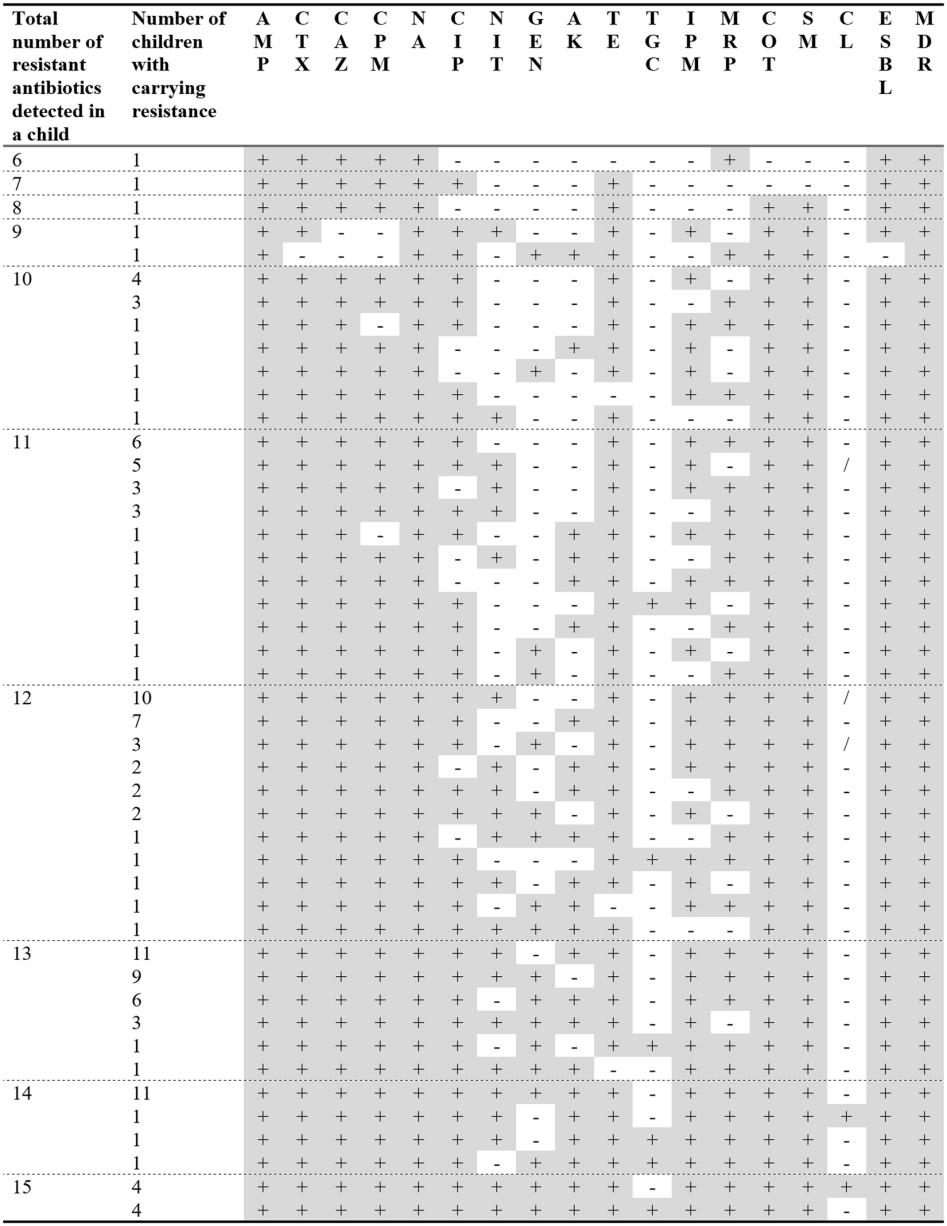
Antibiotic resistance patterns in all 125 children studied. Each row represents a specific resistance pattern found in children at one or more points. +: At least one isolate tested resistant or intermediate resistant, −: All isolates tested susceptible, /: Missing value, AMP: Ampicillin, NA: Nalidixic acid, CTX: Cefotaxime, CAZ: Ceftazidime, CPM: Cefepime, SM: Sulfamethoxazole, COT: Co-trimoxazole, TE: Tetracycline, CIP: Ciprofloxacin; MPM: Meropenem, IPM: Imipenem, NIT: Nitrofurantoin, AK: Amikacin, GEN: Gentamycin, CL: Colistin, TGC: Tigecycline. ESBL: Extended spectrum beta-lactamase, MDR: Multidrug resistance, Count: Number of children with that resistance pattern.

The rates of resistance per child are presented in Fig. 1c. At least one isolate from every child in this study showed ampicillin and nalidixic acid resistance. Resistance rates above 90% were found for cefotaxime, ceftazidime, cefepime, sulfamethoxazole, and co-trimoxazole. The resistance rates were 44.8% (plus an intermediate rate of 36.0%) for meropenem and 37.6% (plus an intermediate rate of 48.0%) for imipenem. Colistin resistance was found in 4.0% of the children, and tigecycline resistance was found in 3.2% of the children (plus intermediate resistance in 4.0%of the children). ESBL resistance was detected in all but one child (99.2%), and MDR was found in isolates from all 125 children. The number of antibiotics to which resistance was found differed considerably, as seen in Fig. 2. Specifically, 13.4% of individual isolates and 3% of stool samples (6 isolates from a single stool sample) were susceptible to all antibiotics. The lowest number of antibiotics to which resistance was found for an individual child (6 isolates from 7 time points) was six. No isolate, stool sample, or child showed resistance or intermediate resistance to all antibiotics. Figure 3 provides the resistance patterns determined by summarising the resistance of all isolates per child. In eight children, all isolates were found to be susceptible only one antibiotic always; in four children, it was tigecycline, and in the remaining four children, it was colistin.

**Figure 4:**
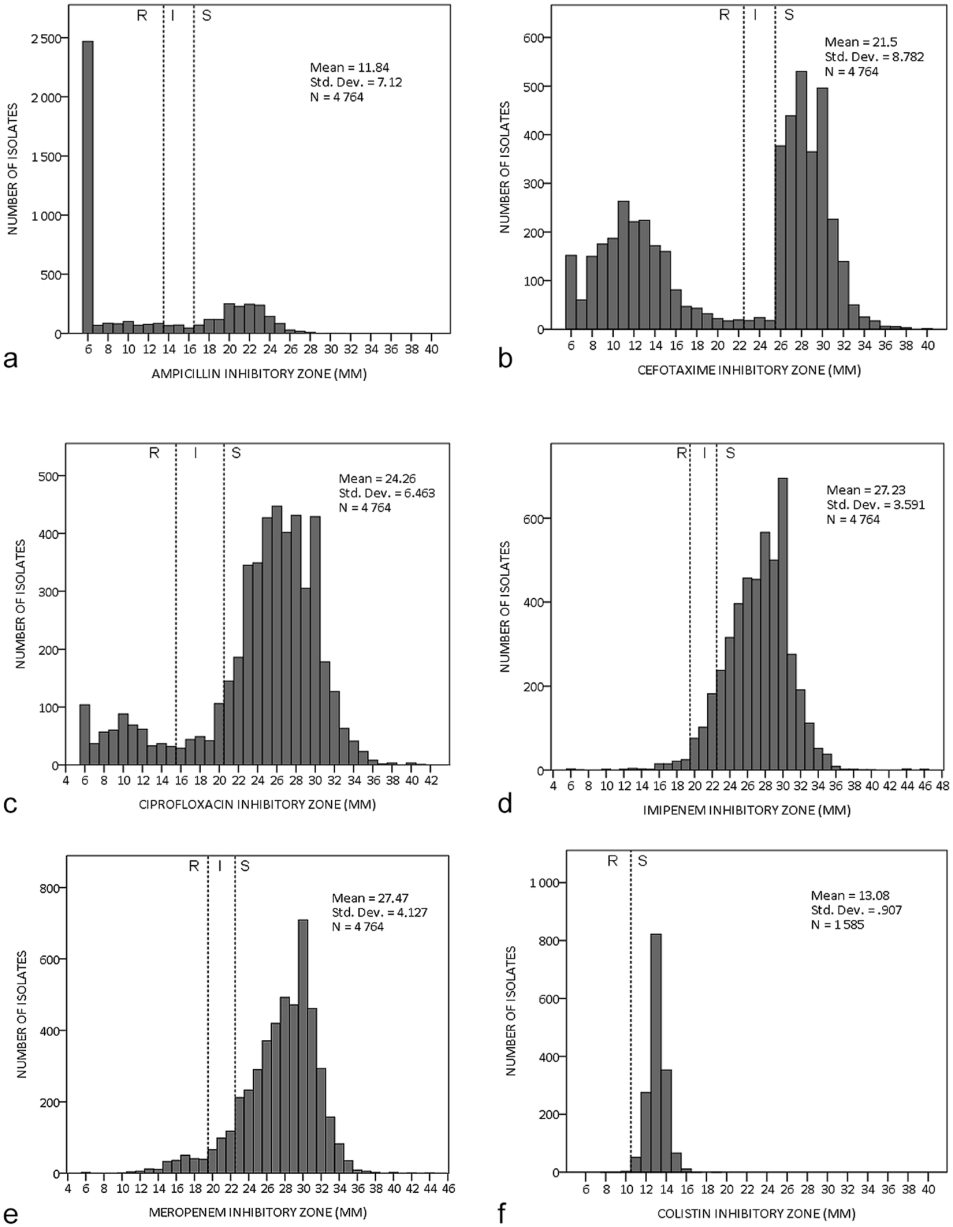
The measured inhibitory zones (mm) of (**a**) ampicillin (**b**) cefotaxime (**c**) ciprofloxacin (**d**) imipenem (**e**) meropenem (**f**) colistin. The boundary values of sensitivity (S), intermediate resistance (I), and resistance (R) of each antibiotic according to CLSI guidelines is marked (colistin does not have a defined zone for intermediate resistance).

The measured inhibitory zones (used to determine resistance) of ampicillin, cefotaxime, ciprofloxacin, imipenem, meropenem, and colistin are presented in Fig. 4. Ampicillin and cefotaxime had bimodal distribution, with a clear division between resistance and susceptibility, and few isolates showed intermediate resistance. Both carbapenems (imipenem and meropenem) had uni-modal distribution, with many isolates showing intermediate resistance. Colistin also had uni-modal distribution, but as per CLSI guidelines, an intermediate resistance zone was not found for colistin.

**Figure 5:**
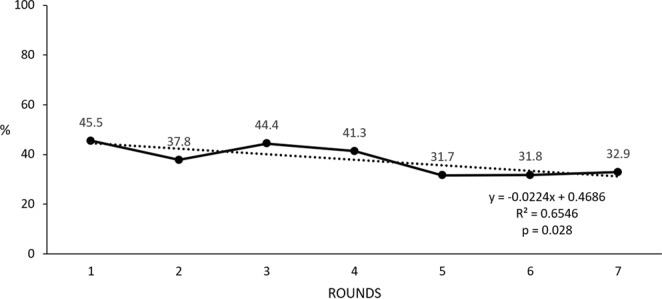
Trend of ESBL prevalence in isolates between time-points (1–7).

The trends of all antibiotics and the ESBL and MDR prevalence during the seven time points were analysed using linear regression. ESBL prevalence showed a significant negative trend (*p* = 0.028; (Fig. 5). No other significant trends could be identified.

### Pathotype and Phylogenetic grouping

The majority (85–100%) of *E. coli* isolates belonged to phylogenetic group A and B1 (considered as commensal) and 0–14.4% belonged to phylogenetic group D (considered as extra-intestinal virulent). The MDR and ESBL producing isolates were mainly (85%) of phylogenetic group A and B1 and the rest, 15% were categorized into group D. No *E. coli* of EHEC, EPEC, STEC, EIEC, EAEC, ETEC and EDEC pathotype were detected while 0–12.6% of *E. coli* were of pathotype ExEPC.

### Correlation with socio demographic factors

Correlation analyses with socio demographic factors identified a significantly lower average prevalence of resistance in stool samples from the female children (5.61 vs 6.13, *p* = 0.043). The children of the families with ration cards (analysed as a proxy variable for poverty) were found to have a significantly higher average prevalence of resistance in their stool samples (5.96 vs 5.36, *p* = 0.029). No significant differences were found in variables related to livestock or personal hygiene; however, correlations of the presence of livestock in the living area (*p* = 0.11) and livestock drinking from a bucket used by household members (*p* = 0.07) with lower resistance were close to reaching significance. Analysis only included variables that potentially affected the prevalence of resistance. All variables included in the analysis are presented in Table 3, along with calculated confidence intervals and *p* values.

**Table 3:** Socioeconomic characteristics of children having resistance to antibiotics per sample per child.

Charatceristics of children	Number of children	Number of resistant antibiotics				
		Mean	Median	Standard deviation	Confid. interval (95%)	p-value
**Age**						
1	62	6.02	5.85	1.36	5.67–6.37	0.34
2–3	63	5.77	5.86	1.51	5.39–6.15	
**Gender**						
Female	57	5.61	5.43	1.47	5.22–6.00	0.04
Male	68	6.13	6.00	1.38	5.80–6.47	
**Family type**						
Nuclear	38	5.92	5.76	1.54	5.41–6.43	0.90
Joint	87	5.88	5.86	1.41	5.58–6.18	
**Ration card**						
Yes	107	5.96	5.86	1.47	5.68–6.25	0.03
No	16	5.36	5.24	0.89	4.88–5.83	
**Water purification method**						
Steel/plastic filter	101	5.97	5.86	1.49	5.68–6.27	0.27
Straining through cloth	20	5.63	5.71	1.19	5.07–6.19	
**Livestock**						
Yes	99	5.88	5.86	1.45	5.59–6.17	0.84
No	26	5.94	5.45	1.44	5.36–6.53	
**Livestock allowed inside living area**						
Yes	56	5.67	5.70	1.42	5.29–6.05	0.11
No	69	6.08	6.00	1.44	5.73–6.42	
**Livestock drinks from bucket used by family members**						
Yes	38	5.55	5.86	1.35	5.11–6.00	0.07
No or no livestock	87	6.04	5.83	1.46	5.73–6.35	
**Cow dung used to clean floors**						
Yes	52	5.99	5.93	1.28	5.63–6.35	0.42
No	71	5.79	5.57	1.54	5.42–6.15	
**Toilet facility**						
Yes	28	6.05	6.93	1.75	5.38–6.73	0.57
No	97	5.85	5.67	1.35	5.57–6.11	
**Substance used to clean hands**						
Soap	99	5.99	5.86	1.46	5.70–6.28	0.14
Mud/ash/only water	26	5.53	5.71	1.34	4.99–6.08	

## Discussion

The study conducted a comprehensive investigation of the antibiotic resistance of commensal bacteria in a community setting, which is a scarcely examined topic. In this study, we explored the prevalence of antibiotic resistance in *E. coli* over 2 years in young children living in a rural area of central India, and we investigated possible correlations with demographic and behavioural factors of the families, which may help explain the spread of resistance. The results of this study fill the knowledge gap of antibiotic resistance in rural India, and they confirmed that the prevalence of antibiotic resistance is substantial for many types of antibiotics.

At any point in time, the human gut is colonised by numerous strains of *E. coli*; the occurrence of some of the strains in the gut is constant, whereas other strains show transient occurrence^21^. Resistance in one isolate will therefore not reflect resistance in all other *E. coli* isolates from the same person at any given time point, and this observation is even less likely over time. The prevalence of resistance per isolate was 63.7% for ampicillin and 48.1% for nalidixic acid; all children in this study possessed *E. coli* isolates resistant to ampicillin and nalidixic acid at one or more time points. Resistance to cefotaxime, ceftazidime, cefepime, sulfamethoxazole, co-trimoxazole, and tetracycline was detected in 14.9–42.5% of isolates, but when summarised, resistance to these antibiotics was found in approximately or more than 90% of the children. The highest prevalence of antibiotic resistance in isolates was observed for ampicillin, which is a common broad-spectrum penicillin included in the 2017 WHO list of essential medicines^22^. A study conducted in 2013 from Ujjain district in India showed a much lower prevalence of 37% in children aged 3–14 years^23^. The result of the present study shows high prevalence of resistance in healthy children as shown in a study on children aged 6–60 months in a rural area of Vietnam^24^. A study in rural Peru and Bolivia revealed a considerably higher prevalence (95%) in healthy children aged 6–72 months^3^. The present study used a different method for determining resistance (following National Committee of Laboratory Standards guidelines); therefore, making the direct comparisons is difficult. The tendency of higher resistance in younger children was also observed in the study conducted in Peru and Bolivia and may reflect a difference in diet, hygiene, and/or antibiotic use. Another explanation for the higher resistance is the rapid increase in the prevalence of antibiotic resistance probably, over the past 5 years in central India.

Notably, in the present study, considerable resistance (4.9% and 2.0% of isolates showed meropenem and imipenem resistance, respectively) was also found for the important carbapenems. Over the entire study period, resistance was detected at least once in 44.8% (meropenem) and 37.6% (imipenem) of the children. In comparison, no resistance to imipenem was found in 529 isolates in a similar study in 3–14-year-old children from Ujjain district, in which meropenem was not tested for^23^. Other studies in children tend to have a very small sample size and to be conducted in hospital settings. A study conducted in a paediatric hospital in Malaysia in 2012 demonstrated that none of 110 strains showed meropenem or imipenem resistance^25^. In a study in Iran, 1% of children with urinary tract infections had isolates resistant to meropenem^26^.

The prevalence of ESBL in *E. coli* widely varies depending on the study location and setting, with generally considerably higher rates in hospital settings than in community settings^27^. In the present study, we detected ESBL in 37.8% of isolates, which is high considering the community setting and the cohort of generally healthy children. Most studies of ESBL prevalence in children have been conducted in hospital or clinical settings, making direct comparisons difficult. Using data for all isolates and at all time points; we found that ESBL prevalence per stool sample was considerably higher (59.8%). In only one child in this study, no ESBL was detected in any isolate, further demonstrating how widespread ESBL is in the study region. This demonstrates how prevalence fluctuates and how it can be related to non-dominant strains.

This study detected low, but some, resistance to colistin and tigecycline, both of which are last-resort antibiotics. Five children showed colistin resistance, and they had the highest overall antibiotic resistance load in this study (resistance to 14.8 antibiotics detected vs the average of 12.1antibiotics). It should be noted that colistin was only tested for during the last three time points. If colistin resistance would have been tested during the entire study period, it may have been found in more children. Tigecycline resistance was found in four children (3.2%), and these children again showed a high load of overall antibiotic resistance (resistance to 13.5 antibiotics vs the average of 12.1antibiotics). In all cases of resistance to colistin and tigecycline, resistance was only identified in one isolate per child; however, one child also had one isolate with intermediate resistance to tigecycline. This result indicates that screening measures using a single isolate may be unlikely to detect colistin and tigecycline resistance. The reason for this may be that colistin and tigecycline resistance exists mainly in non-dominant and/or transient strains. The possibility of error in measurement must also be considered, however, the increased overall antibiotic resistance load in the same children indicate that the results are likely accurate.

One important finding of our study is that the trend of ESBL resistance was found to be significant (*p* = 0.028) and negative when antibiotic resistance prevalence decreased from 45.5% to 32.7% during the 2-year study period. This finding is unexpected, especially considering the substantially lower ESBL in 3–14-year-old children in a previous study conducted a few years earlier in the same province^23^. This finding is in contrast to the general worldwide trend of increasing prevalence. One potential explanation is that the children became older towards the end of the study, which, as mentioned previously, is associated with the lower prevalence of resistance. Another potential explanation is that the intense monitoring of disease in the children during the study may have served as an intervention. A further explanation is that because of the high local ESBL prevalence, health practitioners favoured other classes of antibiotics with lower rates of resistance over third-generation cephalosporin.

The measured inhibitory zones (Fig. 4) revealed different patterns of resistance for different antibiotics. Some antibiotics, such as ampicillin and cefotaxime, had a clear division between resistance and susceptibility, and few isolates showed intermediate resistance. Other antibiotics, especially carbapenems, had a much less obvious difference between resistance and susceptibility. This difference is also significant when interpreting the results, as small measurement error may have greater potential for misclassification for antibiotics with a diffuse boundary.

The gut is the reservoir of both pathogenic and non-pathogenic *E*. *coli* strains and has the ability to transform the pathotypes and exchange antibacterial resistance. Over-the-time, characterization of commensal *E. coli* pathotype in a community-setting shows mainly (0–12.6%) extraintestinal pathotype (ExPEC) with high (85–100%) antibiotic resistance. Although, there was no correlation between over-the-time prevalence and the pathotypes but the high resistance in virulent strains, is a matter of public health concerns. As shown previously, the majority of the *E*. *coli* isolates are categorized as commensal in human (A and B1)^5^.

Another important finding of our study is that the female gender was significantly (*p* = 0.04) related to a lower prevalence of antibiotic resistance. However, some studies have shown lower resistance in female children, whereas other studies have shown lower resistance in male children. The present study found a lower prevalence of resistance (*p* = 0.03) in families possessing ration cards. Possessing a ration card was included in the analysis as a proxy variable for lower wealth. The study in children aged 3–14 years conducted in Ujjain district used education of the mother as a proxy variable, and that study showed higher prevalence of antibiotic resistance with higher wealth^23^. This may be because families with greater wealth have greater access to antibiotics. It can also be reasoned that families with greater wealth can afford cleaner water and better toilet facilities, which would lead to the lower spread of resistance.

In the present study, no significant link was noted between resistance prevalence and socio environmental and behavioural factors, such as the presence of livestock or hygiene practices. It is not unlikely that such an effect still exists, but it may be too small or too complex to be detected using the methodology used in this study. Correlations of the presence of livestock in the living area (*p* = 0.11) and livestock drinking from a bucket used by household members (*p* = 0.07) with lower resistance were close to reaching significance. Interestingly, this result indicates that the presence of livestock may have a protective effect on antibiotic resistance. This result may be because normal flora occupies surfaces; thus, harmful bacteria cannot colonise the surfaces. Strict hygiene measures have been shown to decrease the transmission of resistant organisms in hospital settings, and it has been assumed that the same is true for community settings^28^, although to a lesser extent due to the lower prevalence of antibiotic resistance in community settings.

This study has some limitations; for example, parents or guardians were responsible for collecting stool samples in the morning before the samples were picked up by a research assistant. A further possible concern is the literacy rate of 70.6% in Madhya Pradesh^29^. There is a potential risk that the study participants did not fully understand the information given; however, all information was also given orally. Approximately one-third of the children (n = 41) were not at home during all seven visits by research assistants, and for 19 children, two or more collection days were missed. Summarising resistance in isolates to resistance in stool samples or in children may not be easy, as any initial; erroneously classification in resistance will be multiplied in the results. This, however, is not likely to be a significant problem for antibiotics with a high or intermediate resistance, but it should be considered for antibiotics with very low resistance, such as tigecycline and colistin. The large number of isolates analysed (n = 4,764) and the longitudinal design of the study in which samples were taken at seven time points enabled antibiotic resistance prevalence fluctuation to be investigated. Further, the Kirby–Bauer method of determination (except for colistin) of resistance is well established and robust. Additionally, to minimise human error, two laboratory technicians measured the inhibitory zones of all isolates. Further, gender difference in the antibiotic resistance pattern may be due to a relatively small sample size (125 children), however, considering the number of isolates per child, number of antibiotics studied per child and longitudinal design of the study, it needs attention in surveillance studies for adequate powered validation.

In conclusion, the prevalence of antibiotic resistance in commensal *E. coli* was very high in a community setting in rural India. Although the prevalence of antibiotic resistance in single isolates was high for most type of antibiotics, the prevalence in the children over time was much higher. Resistance to ampicillin, nalidixic acid, cefotaxime, ceftazidime, cefepime, sulfamethoxazole, co-trimoxazole, and tetracycline was detected at least once in 89% of the children or more. ESBL was detected in 124 of the 125 children included in the study, and MDR was detected in all 125children.The female gender and higher wealth was related with the lower prevalence of antibiotic resistance. The results highlight the urgent need for research and interventions in low- and middle-income countries, including India, to control the growing antibiotic resistance crisis.

## Data Availability

The datasets generated during and/or analysed during the study are available from the corresponding author on reasonable request.
